# Autonomous magnetic labelling of functional mesenchymal stem cells for improved traceability and spatial control in cell therapy applications

**DOI:** 10.1002/term.2133

**Published:** 2016-05-06

**Authors:** Richard Harrison, Hareklea Markides, Robert H. Morris, Paula Richards, Alicia J. El Haj, Virginie Sottile

**Affiliations:** ^1^ Wolfson Centre for Stem Cells, Tissue Engineering and Modelling (STEM), School of Medicine University of Nottingham UK; ^2^ Institute of Science and Technology in Medicine Keele University UK; ^3^ School of Science and Technology Nottingham Trent University UK

**Keywords:** mesenchymal stem cell, cell labelling, magnetic microparticle, cell targeting, regenerative medicine

## Abstract

Mesenchymal stem cells (MSCs) represent a valuable resource for regenerative medicine treatments for orthopaedic repair and beyond. Following developments in isolation, expansion and differentiation protocols, efforts to promote clinical translation of emerging cellular strategies now seek to improve cell delivery and targeting. This study shows efficient live MSC labelling using silica‐coated magnetic particles (MPs), which enables 3D tracking and guidance of stem cells. A procedure developed for the efficient and unassisted particle uptake was shown to support MSC viability and integrity, while surface marker expression and MSC differentiation capability were also maintained. *In vitro*, MSCs showed a progressive decrease in labelling over increasing culture time, which appeared to be linked to the dilution effect of cell division, rather than to particle release, and did not lead to detectable secondary particle uptake. Labelled MSC populations demonstrated magnetic responsiveness *in vitro* through directed migration in culture and, when seeded onto a scaffold, supporting MP‐based approaches to cell targeting. The potential of these silica‐coated MPs for MRI cell tracking of MSC populations was validated in 2D and in a cartilage repair model following cell delivery. These results highlight silica‐coated magnetic particles as a simple, safe and effective resource to enhance MSC targeting for therapeutic applications and improve patient outcomes. © 2016 The Authors Journal of Tissue Engineering and Regenerative Medicine Published by John Wiley & Sons Ltd.

## 1 Introduction

Over the past decades, a range of iron oxide‐based magnetic particles (MPs) have been developed for clinical applications in the field of magnetic resonance imaging (MRI) (Gilchrist *et al*., 1957). Superparamagnetic iron oxide nanoparticles (SPIOs) are a specific class of magnetic particles known for their application as T_2_‐weighted negative MRI contrast agents, designed to overcome the inherent low sensitivity associated with MRI (Bulte and Kraitchman, 2004; Pooley, 2005). Magnetic particles composed of either a magnetite (Fe_3_O_4_) or maghemite (*γ*‐Fe_2_O_3_) core (Berman *et al*., 2011; Gupta and Gupta, 2005) surrounded by a biocompatible polymer, such as silica and dextran, have been used for the labelling and identification of cell populations (Kunzmann *et al*., 2011). FDA‐approved iron‐based particles, such as Endorem (also referred to as Feridex) and Resovist, have been used as MRI contrast agents in recent years (Berman *et al*., 2011; Jasmin Torres *et al*., 2011); however, since these products are no longer clinically used, there is a need for validated products offering low toxicity, biocompatibility and chemical stability under physiological conditions (Hofmann‐Amtenbrink *et al*., 2010; Mahmoudi *et al*., 2011).

While the literature on MPs has largely focused on developing particle design, synthesis and characterization (McBride *et al*., 2013), recent studies have also investigated MPs for cell‐based applications beyond MRI imaging, as their applied magnetic fields have been used to develop new approaches to enhance transfection (Pickard *et al*., 2011), induce hyperthermia (Kobayashi, 2011), force *in vitro* aggregation (Fayol *et al*., 2013), enable regenerative therapies (El Haj *et al*., 2012) and activate cell receptor signalling on the cell membrane (Henstock *et al*., 2014). Their small size and magnetic properties, coupled with versatile surface coatings (Gupta and Gupta, 2005), open a range of new approaches which could see MPs enhance existing and future regenerative cell therapies. Such cell‐based approaches require the targeted delivery of functional populations, such as mesenchymal stem cells (MSCs), which have become a resource of prime importance for their skeletal regeneration ability (Caplan, 2007; Quarto *et al*., 2001) but also for their properties of immune modulation (Le Blanc *et al*., 2003), anti‐inflammation (Uccelli, 2008) or trophic secretion (Caplan and Dennis, 2006). MSC‐based therapies for tissue repair require auxiliary approaches which enable *in vivo* tracking, delivery and targeting, in order to monitor and improve the retention of functional cells at the intervention site (Wimpenny *et al*., 2012).

In this study, the suitability of MPs presenting a silica surface with negatively charged silanol groups was investigated for use in human mesenchymal stem cells (MSCs) as a labelling, imaging and manipulation agent. The labelling dynamics and cellular response were analysed with a particular emphasis on markers of cell health, identity and functional potential of the target population, as well as their suitability for cell‐tracking purposes in an articular model. Observations presented here can help refine novel applications of MP labelling and evaluate the resulting health considerations of future MP‐assisted stem cell therapies.

## 2 Materials and methods

All reagents were purchased from Life Technologies, unless otherwise stated.

### 2.1 Human mesenchymal stem cell cultures

A human bone marrow‐derived mesenchymal stem cell line (hMSCs) (France *et al*., 2014; Okamoto *et al*., 2002) was cultured and expanded under standard cell culture conditions (37.5°C, 5% CO_2_) in standard culture medium consisting of Dulbecco's modified Eagle's medium (DMEM) supplemented with 10% v/v fetal bovine serum (FBS), 1% v/v non‐essential amino acids, 1 mm l‐glutamine, 1 mm pyruvate and 1% penicillin–streptomycin. The cells were passaged using trypsin–EDTA. For some experiments, hMSCs stably transfected to constitutively express green fluorescent protein (GFP; gMSCs) following an established protocol (Peister *et al*., 2004) were used under standard cell culture conditions in standard culture medium to enable fluorescence microscopy.

Primary human mesenchymal stem cells (pMSCs) were isolated from human bone marrow aspirate (Lonza, UK). In brief, the bone marrow aspirate was seeded in fibronectin‐coated flasks at a mononuclear cell density of 1.5 × 10^3^ cell/cm^2^ and cultured for 1 week (37°C, 5% CO_2_) in pMSC isolation medium containing low‐glucose DMEM (Lonza Biowhittaker, UK) supplemented with 10% FBS (Lonza Biowhittaker), 1% l‐glutamine (Sigma‐Aldrich, UK) and 1% penicillin–streptomycin (Sigma‐Aldrich). A 50% medium change with fresh pMSC isolation medium was performed after 1 week, followed by a switch 1 week later to hMSC proliferation medium (high‐glucose DMEM supplemented with 10% FBS, 1% l‐glutamine and 1% penicillin–streptomycin). pMSCs were identified as those which had adhered to the tissue‐culture vessel after 14 days in culture.

### 2.2 Cell labelling with magnetic particles (MPs)

hMSCs and pMSCs were labelled with 1000 nm particles composed of a maghemite core with a solid unmodified silica surface, as previously described (Markides *et al*., 2013), using standard (SiMAG) or fluorescently tagged (ScreenMAG‐Silanol) particles, as specified (Chemicell, Germany). In brief, adherent cell populations were incubated with MPs (1–10 μg/ml) in medium for 24 h, using serum‐containing or serum‐free medium (MRI experiments) as specified (for cell‐labelling experiments, standard medium containing 10% FBS was used, unless otherwise stated). The next day, the cells were thoroughly washed with phosphate‐buffered saline (PBS) in order to remove excess particles that may have settled on the surface of the cell layer or flask.

To measure particle uptake by flow cytometry, cells were seeded at 7.5 × 10^3^ cell/ml and ScreenMAG‐labelled for 24 h. The cells were then harvested, centrifuged at 200 × *g* for 5 min and resuspended in 200 μl PBS prior to analysis on a Guava EasyCyte 8HT Flow Cytometer Channel FL2 with InCyte 2.5 Software (Millipore, USA), comparing labelled and unlabelled populations to evaluate percentage uptake based on fluorescent intensity. Analysis was performed using WEASEL (WEHI, Australia), using unlabelled cells as controls to evaluate increased fluorescence. The standard particle concentration used in the study was 10 μg/ml, unless otherwise stated, which was shown to correspond to an intracellular iron load of 20 pg/cell (Markides *et al*., 2013).

### 2.3 Fluorescence imaging of particle uptake

Particle uptake was further evaluated visually using an array of fluorescent cell dyes and fluorescent microscopy to evaluate internalization in relation to cell structure. hMSCs cultured on glass coverslips were labelled with particles and fixed at room temperature for 15 min in 4% v/v paraformaldehyde (PFA; VWR, UK). After permeabilization with 0.1% Triton X‐100 for 5 min following two tPBS washes, cells were stained for actin filaments using a 1:41 working solution of 6.6 μm AlexaFluor® 488 phalloidin in methanol. The slides were incubated in a dark covered container at room temperature for 20 min and then washed twice with PBS, prior to mounting using Vectashield mounting medium (Vector Laboratories, USA). Imaging was performed using a Leica TCS SP2 confocal laser‐scanning microscope (CLSM; Leica Microsystems, Germany).

### 2.4 Prussian blue staining

hMSC cells were grown in monolayer and labelled with 10 μg/ml MPs for 24 h prior to fixing with 4% PFA for 15 min. Immediately prior to addition to the cells, 20% aqueous solution of hydrochloric acid and 10% aqueous solution of potassium ferrocyanide were mixed in equal parts. This staining solution was applied to the fixed monolayer for 5 min and washed three times with PBS. Images were acquired using an Eclipse TS100 inverted microscope (Nikon, Japan).

### 2.5 Transmission electron microscopy (TEM)

To confirm the cellular location of the particles, samples were fixed in 3% glutaraldehyde in 0.1 m cacodylate buffer overnight and post‐fixed in 1% aqueous osmium tetroxide for 30 min. The samples were then dehydrated in a graded ethanol series and infiltrated with Transmit resin (TAAB, UK), then allowed to polymerize overnight at 70°C. Semi‐thin sections were cut (0.5 μm), using a Reichert–Jung ultramicrotome, and stained with 2% toluidine blue. Ultra‐thin sections were cut (70–90 nm) using the same equipment and collected on copper grids, which were then contrasted using 50% methanolic uranyl acetate and Reynolds lead citrate (Robards and Wilson, 1993). Imaging was performed on a FEI Tecnai 12 Biotwin TEM (FEI, USA) with up to 120 kV and ×300 k magnification.

### 2.6 Particle labelling measurement

Flow cytometry was used to measure the level of particle labelling over time. For mitotic arrest, mitomycin C (Sigma Aldrich, UK) treatment was used to halt cell division, using a final concentration of 10 μg/ml for a 2.5 h incubation at 37°C (Nieto *et al*., 2007). The cells were then washed twice with PBS and harvested for use. Mitotically arrested and control cells were cultured over a 7 day period, with cells fixed in 4% PFA for analysis on days 1, 5 and 7. To investigate particle transfer between co‐cultured populations, GFP‐expressing MSCs (gMSCs) labelled with MPs were cultured with unlabelled hMSCs. Both populations were mitotically arrested prior to co‐culture with samples fixed in 4% PFA each day over 7 days, before flow‐cytometry quantification of particle presence and GFP status.

### 2.7 Cell surface marker analysis

hMSCs and pMSCs were assessed for expression of multipotent markers (Dominici *et al*., 2006), performed 24 h after MP labelling (with SiMAG and ScreenMAG, respectively) and 14 days after initial labelling, with repeated passaging and relabelling every 3 days to maintain a high MP level throughout. Cells were harvested with trypsin–EDTA and pelleted by centrifugation for 5 min at 200 × *g* before washing in PBS. The cell pellets were then resuspended in 100 μl PBS supplemented with 5 μl antibodies against CD29 (Abcam, UK), CD105, CD34 and CD73 (AbdSerotec, UK), CD90 and SSEA4 (eBiosciences, USA) for 30 min at room temperature, before two PBS washes and flow‐cytometry analysis.

### 2.8 Cell viability assays

The resazurin metabolic assay was performed to determine metabolic changes, using a working solution consisting of 10% v/v Presto Blue stock solution, prepared according to the manufacturer's instructions. After 45 min of incubation, the fluorescent signals of 100 μl samples were measured at 535 nm excitation and 615 nm emission in triplicate, using an Infinite 200 PRO plate reader and i‐control software (Tecan, Switzerland).

Impact on membrane integrity was assessed using a Live/Dead® AlexaFluor® 488 fixable viability dye. Cells were harvested with trypsin–EDTA and pelleted by centrifugation for 5 min at 200 × *g*, washed twice with PBS and resuspended in 100 μl amine‐reactive dye working solution, consisting of 1% v/v amine‐reactive DMSO stock in PBS. Following 15 min incubation at room temperature, the cells were rinsed twice with PBS and resuspended in 200 μl PBS prior to measurement on a Guava EasyCyte 8HT flow cytometer. Unlabelled cells were used as viable controls and DMSO or PFA fixative treatments provided toxicity controls.

### 2.9 Single‐cell gel electrophoresis (comet) assay

Potential damage to the DNA was assessed with the alkaline comet assay (Seedhouse *et al*., 2006). hMSCs were grown in monolayer and either left unlabelled or labelled with 10 μg/ml or 100 μg/ml SiMAG for 24 h. Following trypsinization, cells were washed once with PBS and resuspended in low melting point agarose (Trevigen, UK) at 10^5^ cells/ml. Comet assay alkaline control cells were used as a positive control for DNA damage (Trevigen). Cell‐containing agarose was immediately spread on comet slides (Trevigen) and left to harden before complete immersion in cell lysis buffer (Trevigen). Lysis was performed overnight at 4°C in the dark. Following this, the lysis buffer was removed and the slides immersed in a UV‐protected electrophoresis tank containing TBE running buffer and allowed to stand for 60 min. Voltage was set at 25 V/CM distance between electrodes and running time at 40 min. Following running, the slides were removed from the buffer and washed three times in distilled water before dipping in ethanol for 1 min and drying overnight. The dry comet slides were stained with 75 μl 0.2% SYBR Green in TBE buffer/agarose droplets. The samples were immediately imaged under a rhodamine filter, using an Olympus BX40 microscope. Comet tails were analysed using Comet Assay III image analysis software (Perceptive Instruments, UK); 50 comet images were obtained from each of the duplicate gel spots and each experimental condition was repeated three times; therefore, 600 images were scored in total for each treatment. The tail moment was used in all analysis.

### 2.10 Mesenchymal differentiation

For differentiation assays, hMSCs were incubated for 21 days in the relevant differentiation media. For osteogenic assays, cells were seeded at 5 × 10^3^ cells/cm^2^ in well plates (Sigma‐Aldrich, UK); the medium was then changed (considered as day 0) every 3 days for 21 days. With either control medium or osteogenic induction DMEM supplemented with 100 nm dexamethasone, 0.05 mm l‐ascorbic acid 2‐phosphate and 10 mm *β*‐glycerophosphate. For adipogenic assays, cells were seeded at 1 × 10^4^ cells/cm^2^ in well plates (Sigma‐Aldrich, UK); the medium was then changed (considered as day 0) every 3 days for 21 days, with either control medium or adipogenic induction high‐glucose (4500 mg/l) DMEM supplemented with 1 μm dexamethasone, 500 μm isobutylmethylxanthine, 10 μg/ml insulin and 1 μm rosiglitazone. For chondrogenic assays, cells were seeded at 37.5 × 10^4^ cells/cm^2^ in flasks for the labelling duration; cells were then detached and 200 μl 1.25 × 10^6^ cells/ml cell suspensions added to 96‐well V‐bottom plates (Nalge Nunc International, USA) and spun at 450 × *g* for 10 min. Following 24 h attachment duration, the medium was then changed every day for 21 days with either control medium or chondrogenic induction high‐glucose (4500 mg/l) DMEM supplemented with 2 mm l‐glutamine, 0.1 μm dexamethasone, 50 μg/ml ascorbic acid phosphate, 1 mm sodium pyruvate, 40 μg/ml Proline, 10 ng/ml TGF*β* and 1× ITS Liquid Media Supplement (Sigma‐Aldrich, UK).

### 2.11 Differentiation assays

Lipid‐containing cells were identified using oil red O (Sheng *et al*., 2007). The cells were washed with PBS and fixed at room temperature for 15 min in 4% v/v PFA. The cells were then washed twice with distilled water and incubated with oil red O working solution added (180 mg/l oil red O in 60% isopropanol/40% distilled water) for 30 min at ambient temperature. The samples were then washed and imaged before extraction of the incorporated stain with isopropanol to measure absorption at 510 nm on an Infinite 200 PRO plate reader and i‐control software (Tecan, Switzerland).

Mineralized nodules were identified using von Kossa staining (Wang *et al*., 2006). Cells were washed with PBS and fixed at room temperature for 15 min in 4% PFA. The cells were then washed three times with distilled water and incubated with 1% silver nitrate in distilled water (Sigma‐Aldrich, UK) under a UV lamp for 15 min. The samples were washed three times with distilled water, incubated for 5 min with 2.5% sodium thiosulphate solution (Sigma‐Aldrich, UK), washed again with distilled water and imaged using an Eclipse TS100 inverted microscope (Nikon, Japan).

Sulphated glycosaminoglycans detected with the dye 1,9‐dimethylmethylene blue (DMMB) were used as an indicator of chondrogenesis. Chondrogenic micromasses were freeze–thawed three times to partially disaggregate them, followed by papain digestion (sodium phosphate 0.1 m, cysteine hydrochloride 5 mm, EDTA 5 mm and papain 45.12 μm in distilled water, pH adjusted to 6.5) overnight at 60°C. Aliquots of digested sample were stained with DMMB dye solution (0.03 m sodium formate, 0.046 mm DMMB, 85.5 mm ethanol and 53 mm formic acid in distilled water), left for 10 min at room temperature and read for absorbance at 540 nm on an Infinite 200 PRO plate reader and i‐control software (Tecan, Switzerland). Aliquots of digested sample were also taken for DNA content analysis with CyQUANT® to allow for normalization. CyQUANT® GR dye/cell‐lysis buffer was added to the samples and incubated for 5 min at room temperature. The samples were analysed on an Infinite 200 PRO plate reader and i‐control software (Tecan, Switzerland).

### 2.12 Directed migration assays

For the vertical migration model, hMSCs were labelled with concentrations in the range 2.5–100 μg/ml alongside unlabelled control cells for 24 h. The cells were then harvested and resuspended to a concentration of 1 × 10^5^ cells/ml. 20 μl drops were deposited, in quadruplicate, on the inside of a multiwell plate lid, which was carefully placed to form hanging drops suspended above humidified wells. A magnetic array constructed from 10 × 3 mm neodymium magnets (2800 gauss; Magnet Expert, UK) was placed above each well, and after 24 h the proportion of cells attached to the undersurface of the lid was evaluated after toluidine blue staining (0.1% for 10 min) and imaging using a 41 Megapixel PureView Zeiss Camera (Nokia, Finland). Quantitative 2D image density analysis was performed using ImageJ (NIH, USA).

For the transmigration assay, SiMAG‐labelled pMSCs (0, 1 and 10 10 μg/ml) were seeded at a concentration of 10^4^ cells/collagen transwell insert (Corning, UK) and allowed to attach for 24 h. The plates were either placed on a magnetic array mimicking a standard 24‐well plate layout or cultured without a magnetic field for 24 h. The collagen layer was then gently removed and the transwell completely washed three times with PBS. Migrated cells located on the underside of the transwell were fixed using 4% formalin for 1 h, stained with DAPI and imaged using a fluorescent microscope. Five independent areas of the well were imaged (top, bottom, left, right and centre) and averaged for each sample.

### 2.13 MRI imaging

To establish the *ex vivo* knee model, chondrocytes were isolated from porcine articular knee cartilage (Staffordshire Meat Packers, Stoke‐on‐Trent, UK) 2 h post‐slaughter, based on a technique adapted from Hayman *et al*. (2006). Cartilage was carefully removed from the upper condyles of the knee, finely diced, weighed and rinsed in PBS and 2% penicillin–streptomycin. After overnight incubation in chondrocyte isolation medium consisting of DMEM/HAM'S F12 (Lonza Biowhittaker, UK), 2% penicillin–streptomycin, 50 μg/ml sterilized ascorbate (Sigma‐Aldrich, UK), 1 mg/ml clostridial collagenase (Sigma‐Aldrich) and 0.1 mg/ml DNAse (Sigma‐Aldrich), the digested cartilage suspension was filtered through 100 μm cell strainer and centrifuged at 600 × *g* for 10 min. Chondrocytes were seeded at 2 × 10^4^ cells/cm^2^ and cultured in chondrocyte proliferation medium (DMEM/HAM'S F12 supplemented with 10% FBS, 1% l‐glutamine and 1% penicillin–streptomycin).

The *in vitro* MRI visibility threshold of SiMAG‐labelled cells populations (0, 1, 5, 10 and 100 μg/ml) was investigated at varying cell densities (5 × 10^5^, 10^5^ and 10^4^) in 2 mg/ml rat tail type I collagen gel (BD Biosciences, UK). The samples were then imaged using a 2.3 T Brucker animal scanner (NTU, Nottingham, UK), with MSME sequences using 1000 ms repetition time, 10.25 ms echo time with eight echoes, and a matrix size of 256 × 192 with a spatial resolution of 0.469 × 0.625 mm.

*Ex vivo* imaging was carried out using a cadaveric porcine knee model of articular cartilage damage to assess the visibility threshold of MP‐labelled cells in a clinically relevant model of autologous chondrocyte implantation (ACI) to treat cartilage damage (Chiang *et al*., 2005). Pig legs were processed to remove all surrounding tissue, using a surgical scalpel. Once the knee had been isolated, the patellar tendon was sliced and the patella pulled back to reveal the articulating ends of the femur and tibia. The knee was then bent to fully expose the upper condyles, and cartilage flaps were created (1.5 × 0.5 × 1.5 cm) across the upper condyles of the knee. Two defects were created on each condyle (left and right), at least 0.5 cm apart. MP‐labelled cells were suspended in a collagen type 1 gel solution (4.5 mg/ml) and injected within the defect while the knee was in the bent upright position, taking care to ensure no bubbles or leakage occurred. After the gels had set (1 h, 37°C) the leg was straightened and the patella replaced and securely bandaged to prevent excess movement, before storage at –20°C until imaging at the MARIARC centre (Liverpool University), using a Siemens Symphony 1.5 T scanner. One day prior to MR imaging, the samples were defrosted, placed within a circularly polarized extremity coil, and double‐echo steady‐state (DESS) sequences were applied, in agreement with MRI scanning conditions implemented in the imaging and diagnosis of human knee pathologies.

### 2.14 Statistical analysis

Statistical analysis was in the form of ANOVAs performed using GraphPad PRISM (GraphPad Software, USA). Tukey's *post hoc* analysis was performed to determine the significance between subgroups of the analysed population. Significance was shown as *p < 0.05, **p < 0.01, ***p < 0.001 and ****p < 0.0001.

## 3 Results

### 3.1 Cytocompatibility study

**Figure 1:**
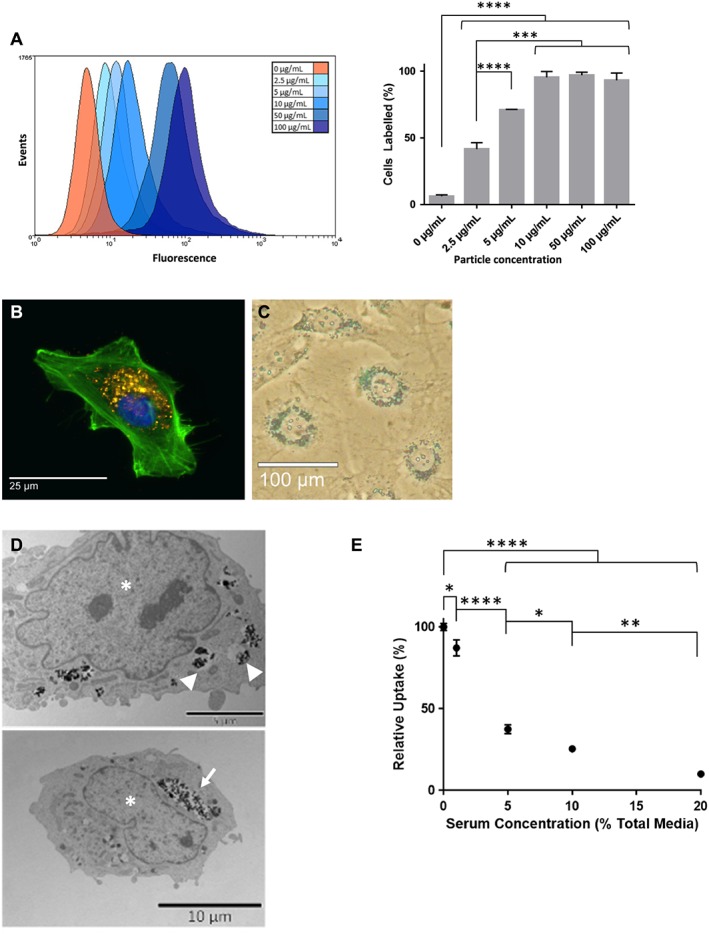
MSCs labelled with fluorescently labelled MPs, analysed using both flow cytometry and microscopy. (A) Flow cytometry analysis (left) and corresponding quantification (right), showing increased labelling with increasing MP concentrations (***p < 0.001, ****p < 0.0001; *n* = 3). (B) Fluorescence imaging of hMSCs labelled with 10 μg/ml particles, showing MPs (yellow), cell outline (phalloidin; green) and nuclear counterstain (Hoechst 33342; blue); bar = 25 μm. (C) Prussian blue staining, highlighting internalized iron‐rich MPs within the cell; bar = 100 μm. (D) TEM imaging of MPs, showing that internalized particles are contained within vesicles (arrowhead), which merge into larger vacuoles (arrow) near the nucleus (*); bar = 5 μm (top) and 10 μm (bottom). (E) Negative effect of serum concentration on the efficiency of MP uptake, measured at 24 h after labelling (*p < 0.05, **p < 0.01, ****p < 0.0001; *n* = 3). [Colour figure can be viewed at wileyonlinelibrary.com]

To evaluate the capacity of MSCs to take up MPs, monolayer cultures were incubated overnight with various concentrations of particles. Particle uptake in hMSCs following a 24 h incubation period with MPs was analysed by fluorescence microscopy and flow cytometry (Figure 1).

Incubation with increasing doses of MPs led to a proportional increase in the fluorescence signal measured for hMSCs (Figure 1A). Time‐lapse microscopy (see supporting information, Video S1) and fluorescence microscopy (Figure 1B) confirmed particle uptake while the cells retained morphology after labelling. Prussian blue staining allowed visualization of the iron‐containing particles present within the cells (Figure 1C). TEM imaging confirmed the presence of MPs within the cytoplasm and highlighted their localization to vesicles found to congregate around the nucleus (Figure 1D). The efficiency of MP uptake was compared under different serum concentrations using flow cytometry, which demonstrated a dose‐dependent negative effect of serum on cell labelling (Figure 1E).

**Figure 2:**
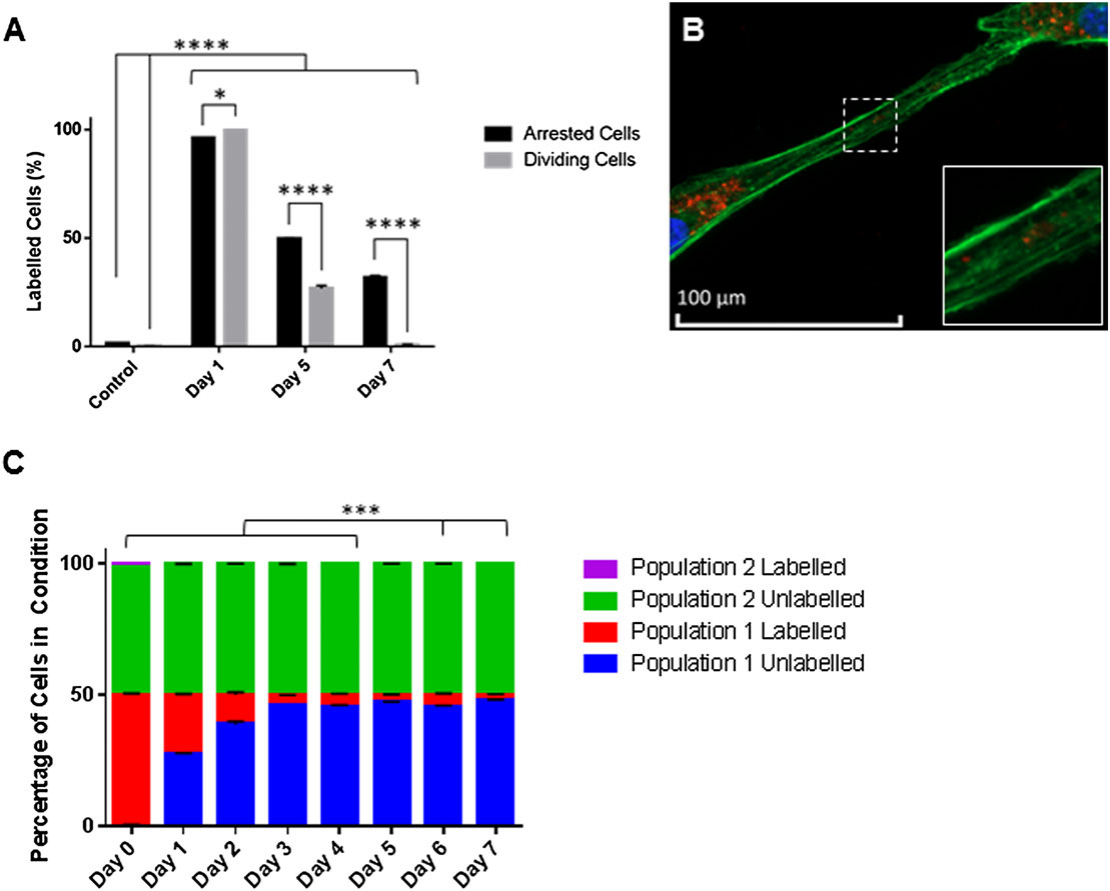
Kinetics of MSC particle retention after labelling with 10 μg/ml particles. (A) Flow‐cytometry analysis of MPs in labelled cells up to 7 days after labelling, showing gradual decrease in dividing cell populations (light grey), while particle dilution is reduced by mitomycin C‐mediated inhibition of cell division (dark grey) (*p < 0.05, ****p < 0.0001; *n* = 3). (B) Fluorescence microscopy of phalloidin staining (green) with DAPI counterstain (blue), showing rare particles (red) detected in cell processes. (C) Distribution of MPs between a labelled (population 1, gMSCs) and unlabelled (population 2, hMSCs) MSC population, analysed by flow cytometry over 7 days of co‐culture, showing no evidence of secondary particle uptake; statistical analysis, showing labelling of population 1 between days 0–4 and 6 compared to day 7 but no statistically significant labelling present in population 2 on any day (***p < 0.001; *n* = 2). [Colour figure can be viewed at wileyonlinelibrary.com]

Following uptake, particle retention was analysed over time in culture (Figure 2). In dividing hMSCs, MPs were found to be progressively diluted until day 7, when they were not detected (Figure 2A). In mitotically arrested cells, however, the particles were retained more efficiently and showed a significant retention compared to untreated cells at day 7, suggesting that the MP load might be divided between daughter cells. Observation of labelled cells showed the occasional presence of some isolated particles within cell projections (Figure 2B).

To investigate the fate of the particles over time, a co‐culture experiment was set up to examine whether MPs may be transferred between labelled and unlabelled hMSC populations (Figure 2C). GFP‐expressing MSCs (gMSCs) labelled with MPs were mixed with control unlabelled hMSCs, and over 7 days in co‐culture cells were analysed by flow cytometry to evaluate the proportion of MP‐containing cells within each MSC population. While a decrease in the percentage of MP‐containing gMSCs was seen over time, there was no detectable appearance of MP‐containing cells in the unlabelled hMSC population over 7 days.

**Figure 3:**
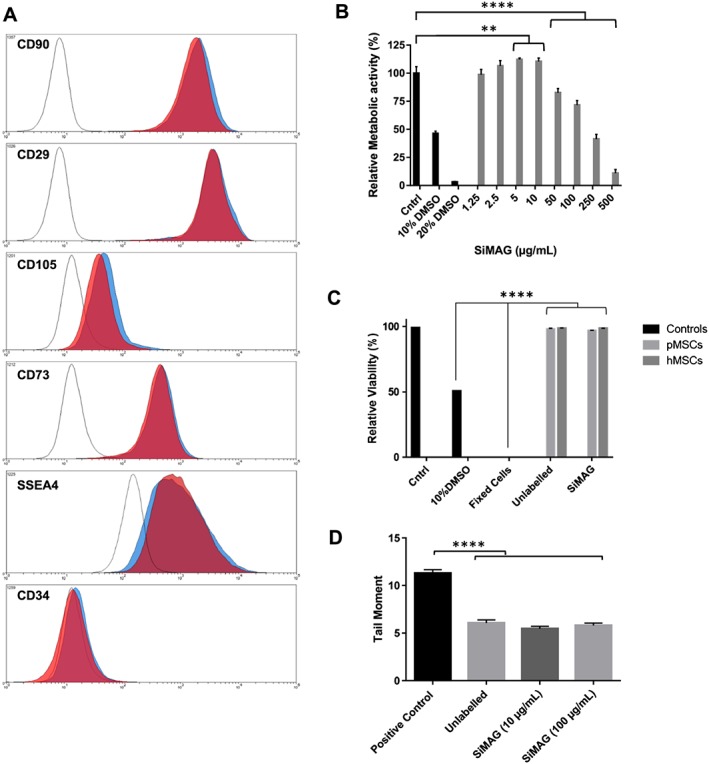
Cell integrity assessment after particle uptake. (A) MSC marker identity analysed by flow cytometry, demonstrating no discernible change in hMSC marker expression following particle labelling (red, 10 μg/ml) compared to unlabelled cells (blue) and the isotype control (grey). (B) Metabolic activity assessed through a resazurin analogue (Presto Blue®) at 24 h, demonstrating no significant negative effect of particle uptake at therapeutic doses (up to 50 μg/ml) when compared to unlabelled controls and DMSO‐mediated toxicity (**p < 0.01, ****p < 0.0001; *n* = 3). (C) Cell membrane integrity assay, showing stable membrane integrity 24 h after labelling with MPs (10 μg/ml); statistical significance calculated compared to DMSO‐treated or fixed cells (****p < 0.0001; *n* = 3), no statistically significant difference between treatment groups. (D) DNA integrity analysed using the comet assay, showing no statistically significant DNA damage in labelled cells at 10 and 100 μg/ml; statistical significance between induced damage (positive control) and other conditions (****p < 0.0001; *n* = 680), no significant difference between unlabelled and MP‐labelled conditions. [Colour figure can be viewed at wileyonlinelibrary.com]

The effect of MP exposure on cell identity was analysed through surface marker analysis and cell integrity assays (Figure 3). Using markers associated with MSCs, comparable positive expression of CD90, CD105, CD73, SSEA4 and CD29, with negative expression of CD34, was confirmed between labelled and unlabelled control populations 24 h after labelling (Figure 3A). Cultures exposed to serial MP labelling every 3 days for 14 days to maintain maximum dose similarly demonstrated retained marker expression, confirming that exposure to MPs did not elicit a significant change in marker identity (see supporting information, Figure S1).

The effect of MP exposure was further investigated through metabolic assays of MSCs labelled with increasing doses of SiMAG MPs, using a resazurin‐based dye, Presto Blue. The data gathered demonstrated a slight increase in metabolic activity at low particle doses and a decreased metabolic activity associated with very high doses 24 h after labelling (Figure 3B). This increased metabolic activity at low MP doses appeared to be lost 48 h after labelling (data not shown). Cell membrane integrity, assessed using flow cytometry, indicated that no effect of MP labelling could be detected 24 h (Figure 3C) after labelling, for either pMSCs cells or hMSCs.

Since MPs were found to accumulate close to the nucleus, their possible effect on cellular DNA was examined using the comet assay, which provides a sensitive measure of DNA damage throughout the population (Figure 3D). No statistically significant increase in DNA damage was observed at 10–100 μg/ml when compared to unlabelled MSC controls (*p* > 0.05).

### 3.2 Application of MSC labelling for regenerative medicine

**Figure 4:**
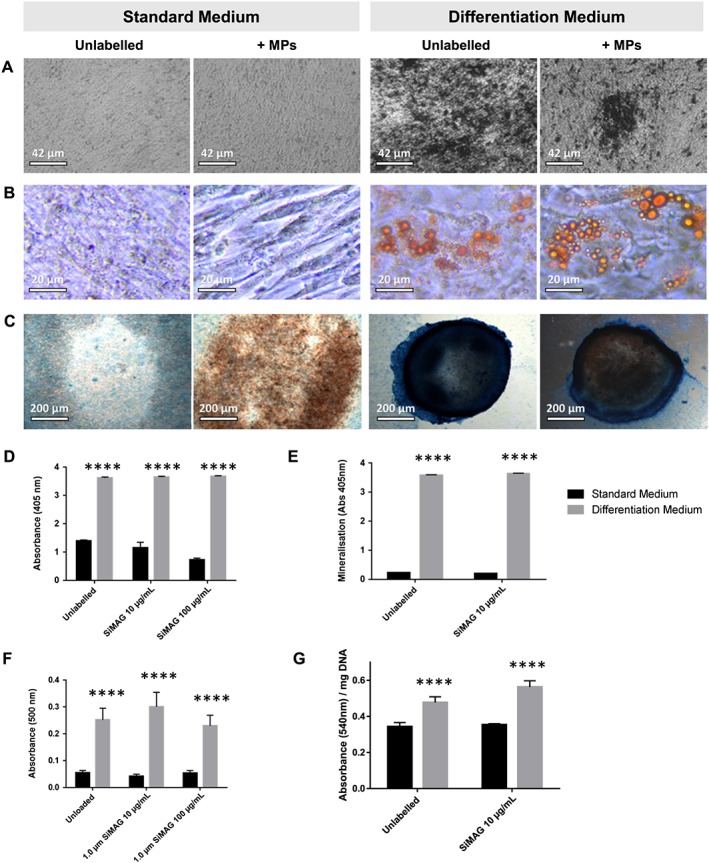
MSC differentiation in the presence or absence of MPs. (A–C) Differentiation potential under (left panel) standard culture medium or (right panel) differentiation treatment of the hMSC populations towards (A) osteogenic, (B) adipogenic and (C) chondrogenic lineages, monitored by von Kossa, oil red O and Alcian blue staining, respectively: MP‐labelled cell populations (10 μg/ml) were compared to unlabelled populations, with no detectable decrease in differentiation *in vitro*. (D, E) Quantitative assessment of osteogenic response performed at 7 (alkaline phosphatase activity) and 14 (alizarin red S extraction) days, showing statistically significant response to induction medium (grey bars) compared to untreated controls (black bars). (F) Adipogenic induction was measured using oil red O extraction, demonstrating no statistically significant change in lipid accumulation at either concentration. (G) Chondrogenic response, assessed using the DMMB assay normalized to DNA content, showing increased GAGs in both unlabelled and labelled populations compared to their standard medium‐treated equivalents (****p < 0.0001; *n* = 5). [Colour figure can be viewed at wileyonlinelibrary.com]

After establishing the cytocompatibility of particle labelling, the efficiency of the differentiation response obtained under various culture conditions was evaluated in MSCs. hMSCs, either unlabelled or labelled with SiMAG, were treated with osteogenic, adipogenic and chondrogenic media for 7 and 14 days to measure their response with and without MP exposure (Figure 4). After 21 days in culture with relevant differentiation media, histological staining (Figure 4A–C) showed successful responses, as detected through mineral deposition (von Kossa staining for the osteogenic condition), lipid accumulation (oil red O staining for the adipogenic condition) and glycosaminoglycan (GAG) production (Alcian blue staining for the chondrogenic condition). Subsequent quantitative assays revealed no significant difference between unlabelled and MP‐labelled cell populations for the osteogenic alkaline phosphatase activity and alizarin red O assays (Figure 4D, E) or for adipogenic oil red O staining (Figure 4F). Quantitation of GAG formation in response to chondrogenic treatment (Figure 4G) showed no detrimental effect of MP labelling, which produced a slight detectable increase in signal compared to unlabelled controls. These data demonstrate no reduction in differentiation capacity following particle labelling.

**Figure 5:**
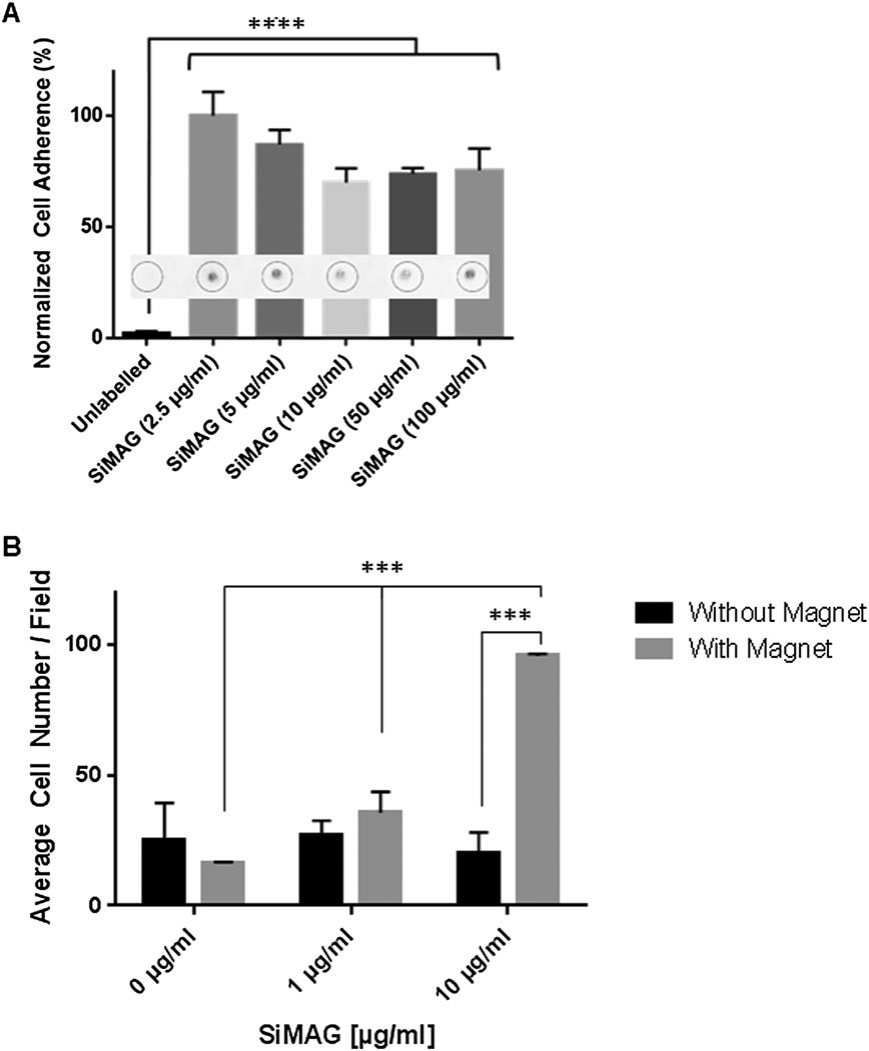
Migration of SiMAG‐labelled MSCs *in vitro*. (A) Hanging drops prepared with cells labelled with increasing MP concentrations were incubated in the presence or absence of magnets placed on the upper side of the lid; after 24 h, surface areas covered by cells recruited to the surface of the lid (inserts) were imaged and measured (****p < 0.0001; *n* = 4). (B) MSCs labelled with MPs (0, 1 and 10 μg/ml) over a 24 h period within a collagen transwell system and exposed to a magnet for 24 h; migrated cells counted as the average of five fields of view on the underside of each transwell (***p < 0.001; *n* = 3)

The iron core present in SiMAG particles makes them susceptible to magnetic forces, a feature potentially beneficial for novel tissue‐engineering approaches. In order to test whether MSC labelling with SiMAG could provide added control over the behaviour of the cells, a migration assay was run to measure the cellular response *in vitro* (Figure 5). When exposed to a permanent magnet located above the samples for 24 h (Figure 5A), labelled cells displayed a significant higher vertical migration towards the magnet when compared to unlabelled samples, which failed to migrate and adhere. When observing cells recruited to the lid in response to magnet exposure, cells labelled with higher MP concentrations appeared to aggregate over a smaller, more defined area at the centre of the lid, rather than spread over a larger surface area, as seen at the lower dose (2.5 μg/ml), possibly due to a stronger cell response at the point of highest field strength, but this 3D aggregation could not be accurately quantified using this 2D adherence assay.

To confirm the magnet‐assisted migration response of cells labelled using particle concentrations previously shown to maintain cellular integrity, a further experimental model was used, in which MSCs were seeded onto a porous collagen scaffold and exposed to a magnetic field (Figure 5B). Cells labelled with 10 μg/ml MPs showed a significantly enhanced migratory capacity compared to unlabelled cells (*p* < 0.001).

**Figure 6:**
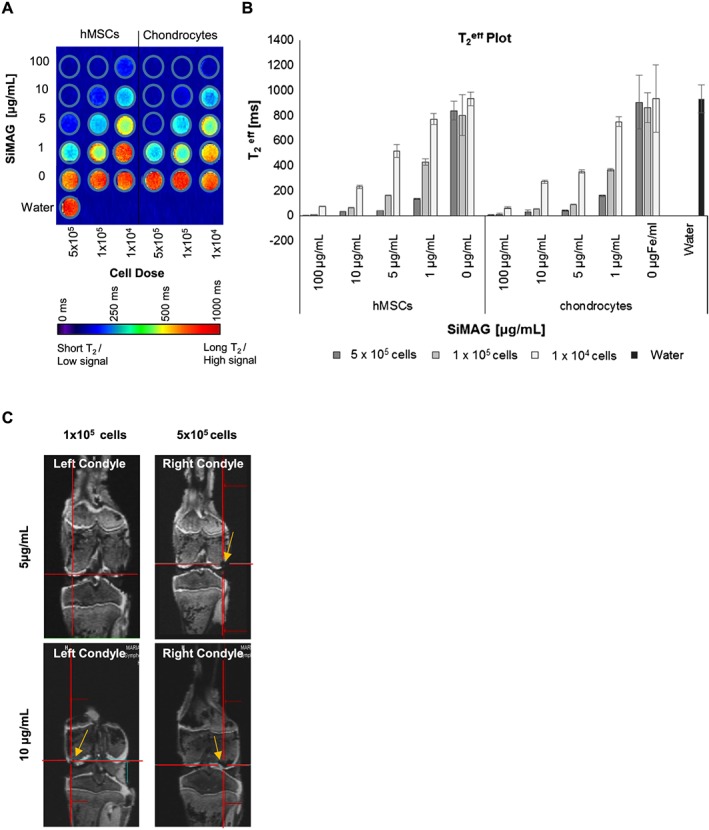
MRI tracking of SiMAG‐labelled hMSCs and chondrocytes. (A, B) Increasing MP concentrations (1, 5 and 10 μg/ml) and cell doses (10^5^ cells, 5 × 10^5^ cells), showing the MRI visibility threshold of labelled MSCs presented as (A) a T_2_^eff^ map and (B) a corresponding T_2_^eff^ plot. (C) Coronal DESS image of labelled chondrocytes implanted in a porcine knee joint (left condyle 10^5^ cells, right condyle 5 × 10^5^ cells), analysed by MRI using 5 μg/ml (upper panel) and 10 μg/ml (lower panel) MP concentrations, showing hypo‐intense regions of signal voids (yellow arrows); red lines highlight the region of interest (ROI). [Colour figure can be viewed at wileyonlinelibrary.com]

SiMAG particles can also act as potential contrast agents, which could allow post‐delivery of cellular therapies for applications such as cartilage repair. In such approaches, an exogenously expanded cell population would be delivered to a discrete site, where it would need to be retained in order to promote local tissue repair (El Haj *et al*., 2014). The ability to image and monitor the implanted cells would allow monitoring of the therapy over time (Markides *et al*., 2013). In order to identify the variables for cell tracking after labelling, the MRI visibility thresholds required in terms of particle concentration and cell number were established first *in vitro* and then in a preclinical large animal model of cell injection (Figure 6). When monitored *in vitro* (Figure 6A), SiMAG‐labelled MSCs and chondrocytes were clearly detectable by MRI with significant dose‐dependent contrast when using doses in the range 10^4^–0.5 × 10^6^ cells. T_2_^eff^ (Figure 6B) was seen to decrease with increasing cell numbers and particle concentrations corresponding to an increasing Fe content. A minimum visibility threshold of 5 μg/ml used with 5 × 10^5^ labelled cells was identified *in vitro*. The detectability of MSC and chondrocyte cell populations after SiMAG labelling was found to be comparable in this model.

To further evaluate imaging capability *in vivo*, labelled cell populations were resuspended in a collagen type I gel, a substrate widely used in cartilage tissue engineering (Deponti *et al*., 2013), injected into a porcine knee model (Chiang *et al*., 2005) and MR‐imaged using specific T_2_‐weighted sequences (Figure 6C). In this clinically relevant model, the effect of particle concentration on MRI detection was analysed by implanting varying cell doses (10^4^, 10^5^ and 5 × 10^6^) of SiMAG‐labelled cells to determine the visibility threshold, using two particle concentrations (5 and 10 μg/ml). A combination of 10^5^ cells labelled with 10 μg/ml was found to provide suitable contrast to enable graft detection by MRI within the host tissue.

## 4 Discussion

### 4.1 Efficient unassisted labelling of hMSCs

Magnetic particles offer interesting properties for a multitude of biological and biomedical applications. Superparamagnetic iron oxide nanoparticles have already demonstrated clinical efficacy and safety for MRI imaging (Colombo *et al*., 2012) and are now being investigated for more advanced theranostic applications for cell tracking and manipulation (Corot *et al*., 2006; Hu *et al*., 2014). MRI agents are developed to be bio‐inert, in order to minimize interaction with the cells within the body. Conversely, cell‐labelling agents must interact with the cell of interest to enable labelling without impacting upon its normal function. Thus, characterization of cell–MP interactions needs to be thoroughly assessed for advanced applications in cell‐based therapies. In this study, we evaluated the suitability of commercially available 1 μm silica‐coated particles as a non‐toxic labelling agent for cell tracking and manipulation towards both *in vitro* and *in vivo* applications.

24 h of incubation of MSCs with MPs was found to allow efficient labelling of the cell population, with > 95% of cells labelled at 10 μg/ml, as measured by flow cytometry. This is in line with previous publications describing near‐100% cell labelling using visual inspection following Prussian blue staining or iron measurements (Balakumaran *et al*., 2010; Kostura *et al*., 2004; Liu *et al*., 2011; Markides *et al*., 2013; Pawelczyk *et al*., 2006). A dose of 10 μg/ml was selected as the standard labelling concentration for MSCs, which was comparable to other reports (7 μg/ml, Liu *et al*., 2011; 25 μg/ml, Kostura *et al*., 2004).

Cell labelling experiments demonstrated rapid uptake of MPs into MSCs, resulting in efficient cell labelling without the need for an added chemical carrier. Previous studies have suggested that stem cell populations may benefit from assisted MP uptake through cellular targeting (Lewin *et al*., 2000) or the use of transfection agents, including polyethylenimine, protamine sulphate and polylysine (Arbab *et al*., 2004; Balakumaran *et al*., 2010; England *et al*., 2013; Jing *et al*., 2008a; Kostura *et al*., 2004; Schafer *et al*., 2010). Interestingly, past reports have mentioned inefficient uptake by rat MSCs (Jing *et al*., 2008b) and undetectable uptake with human MSCs (Kostura *et al*., 2004) when different particles were used alone. In contrast, our results confirm highly efficient uptake of the SiMAG particles in the absence of any additional facilitator, in line with observations carried out in other stem cell populations (Chen *et al*., 2013). Particle surface modifications influence the characteristics of size, charge, toxicity and degradability of the particle (Li *et al*., 2013) and have previously been reported to influence particle–cell interactions (Gupta and Gupta, 2005; Sakhtianchi *et al*., 2013; Zhao *et al*., 2011). The SiMAG particles used here were silanol‐coated, presenting an activated Si–OH surface arrangement. One of the main benefits of the silanol surface is a high colloidal suspension stability, even in high volume fractions, through pH changes and electrolyte disturbances (Mulvaney *et al*., 2000), all of which are likely to occur to some degree during application in a physiological environment. When silanol‐coated MPs come into contact with the membrane, their association with the phosphatidyl choline‐rich regions of the membrane (Zhao *et al*., 2011) is thought to elicit a membrane‐wrapping effect as other regions associate with the rigid curvature of the silanol surface. The subsequent entry of the MPs is dependent upon the energy released through the exothermic membrane‐wrapping effect and the energy required to bend the membrane around the MP completely. In this situation, the dense nature of these MPs is considered to decrease the energy required for deformation of the membrane, thus facilitating engulfment (Zhao *et al*., 2011), as suggested by the report that larger MPs are more thermodynamically favourable for endocytosis (Slowing *et al*., 2009). Surface properties of the particles may also influence their interaction with natural proteins from serum (Wiogo *et al*., 2011). The data presented here further demonstrate that the presence of serum diminishes SiMAG particle labelling in a dose‐dependent manner, potentially due to diminished accessibility of the surface silanol groups to the membrane, in line with previous reports supporting cell loading in serum‐free conditions (Wilhelm and Gazeau, 2008).

The efficient uptake of the SiMAG particles allowed labelled stem cell populations to be monitored through both their iron content and fluorescent analysis techniques. Particles appeared to cross the extracellular membrane, possibly through membrane wrapping and engulfment, as previously described for silica particles (Zhao *et al*., 2011), although the exact nature of this process requires further examination. Once inside the cell, the particles accumulated at a central location inside endosome‐like structures proximal to the nucleus, and no particle was observed inside the nuclear space, likely due to their micron size and contrary to what has been reported for particles <70 nm (Chen and von Mikecz, 2005). Such an intracellular particle distribution has previously been observed in MSCs (Chang *et al*., 2012; Neuberger *et al*., 2005) and other cell types (Robert *et al*., 2010b; Sun *et al*., 2012; Wilhelm and Gazeau, 2008).

### 4.2 Cellular compatibility

Whilst previous studies have described the use of different particle types for cell labelling, few have focused on the potential implications of MP labelling for MSC health and function. Among these, most reports have investigated the biocompatibility of smaller MPs used with an auxiliary labelling reagent (Arbab *et al*., 2004; Balakumaran *et al*., 2010). Here, the suitability of SiMAG labelling for human MSCs was carefully examined through a range of parameters reflecting the integrity and cell health of labelled MSCs. Previously published studies on MP cytocompatibility have largely relied on the assessment of cell morphology combined to MTT/MTS assays; however, these have demonstrated questionable reliability for particle and nanomaterials studies (Laaksonen *et al*., 2007). A resazurin‐based metabolic measurement was therefore selected here, and indicated a slight increase in metabolic activity after particle labelling at low doses of particle uptake. This mild effect, which has been mentioned in different experimental conditions, could be linked to homeostatic mechanisms increasing lipid membrane synthesis in the cell to compensate for extracellular membrane disturbance associated with particle internalization (Kowalski *et al*., 1972; McNeil and Steinhardt, 1997). Similarly, MSC surface marker expression analysed before and after labelling showed that both primary and established MSCs retained their cell identity (Dominici *et al*., 2006). This matches observations reported for different models and labelling conditions, which reported no significant change in MSCs (Balakumaran *et al*., 2010), and similar stable marker expression in haematopoietic stem cell populations (Arbab *et al*., 2004).

Although previous studies have suggested good MP cytocompatibility for cell cultures (Budde and Frank, 2009; Heymer *et al*., 2008; Li *et al*., 2013), some observations using small‐sized MPs (60 nm) have described changes in MSC migration, colony‐formation efficiency and even differentiation after particle labelling (Schafer *et al*., 2009). Similar MP concentrations have also been reported to cause significant toxicity in neuronal and glial cells, while they did not appear to affect other cell types, such as cardiomyogenic and pancreatic cells (Laurent *et al*., 2012; Mahmoudi *et al*., 2011). It is therefore important to evaluate the toxicity of each MP labelling protocol to be used in the target cell model for the application considered. MP‐related toxicity may arise from the leaching of ions from the metal core and the biodegradation polymer coating, which could cause oxidative stress (Kim *et al*., 2011) through the leaching of metal ions from the core, or the release of oxidants by enzymatic degradation of the MPs (Mahmoudi *et al*., 2012). Although iron can be metabolized in the human body (Berry, 2005; Bulte *et al*., 2009; Henning *et al*., 2009; Ju *et al*., 2006; Kim *et al*., 2010a), high quantities of Fe can impair viability and normal cell function (He *et al*., 2007; Li *et al*., 2013), underlining the need for a suitable balance between high Fe incorporation and safe cell function. Particle concentrations in the range 2.8–400 μg/ml have been reportedly used for *in vivo* tracking (Farrell *et al*., 2009; He *et al*., 2007; Jing *et al*., 2008b; Kim *et al*., 2010b). The particle concentration chosen for this study (10 μg/ml), which was selected within the lower end of this range, showed no significant effect on cell viability or on the level of DNA damage in the MSC population, as measured by the comet assay. This was true even for higher concentrations (100 μg/ml) and is in line with other studies that have shown low toxicity of both Fe_3_O_4_‐ and Fe_2_O_3_‐based particles (Karlsson *et al*., 2009).

In addition to preserving the health of labelled cell populations for future cell therapies, maintaining their functionality is equally critical if they are to deliver a therapeutic effect. Reports published to date have provided mixed results for the impact of MPs on MSC differentiation. While a majority of studies reported no significant change based on histological or molecular assays, some negative effects on chondrogenesis have been observed (Bulte *et al*., 2004; Kostura *et al*., 2004). To examine the suitability of SiMAG‐labelled MSCs to fulfil a therapeutic role, we examined their ability to differentiate into the osteogenic, adipogenic and chondrogenic lineages and found it to be maintained when examined both qualitatively and quantitatively. Bone nodules and lipid droplets were present in their respective cultures, with no statistically significant differences between unlabelled and labelled cell populations. Chondrogenic differentiation yielded micromass pellets demonstrating positive staining of glycosaminoglycans (GAGs) for both control and MP‐labelled cultures. Closer examination revealed an increase in GAGs measured in MP‐labelled pellets compared with the unlabelled samples, which could be due to more efficient centrifugal aggregation of the MP‐labelled cells, as observed in our culture, since this is an important experimental parameter for the establishment of micromass cultures.

### 4.3 Control of target cell populations

The possible dilution of the particle load by either exocytosis or cell division represents an inherent limitation of MPs and MRI‐based tracking in cell‐based therapies, which could be of concern in long‐term animal studies. MSC labelling was detected here during a 7 day period in the case of dividing cell populations, beyond which the intracellular particle concentrations returned to control levels. However, this was not solely dependent upon cell division, as previously observed with smaller particles (Kim *et al*., 2012; Wilhelm and Gazeau, 2008), since non‐dividing populations also demonstrated particle loss, albeit at a reduced rate. Arrested cells still demonstrated around 30% labelling 7 days after labelling, suggesting the occurrence of particle release or biodegradation in addition to mitotic dilution. Particle loss has been described as size‐dependent, with smaller particles reportedly exocytosed at a faster rate than larger particles (Sakhtianchi *et al*., 2013). Interestingly, this would fit with the observation of MP‐labelled mouse MSCs implanted subcutaneously, showing halving of the MRI signal over 3 days and over one‐third of the initial signal detected by day 7 (Liu *et al*., 2011). Berman *et al*. (2011) suggested particle decrease to be an indicator of viable cells, as non‐viable cells may also retain the particles due to an inability to divide or actively exocytose.

It is unclear whether magnetic labelling of MSCs may be associated with particle loss *in vivo*, and whether this may lead to subsequent unspecific labelling through secondary particle uptake by an unintended population. Results from our co‐culture model combining labelled and unlabelled MSCs showed that the gradual loss of particles from a labelled cell population did not result in any significant uptake by neighbouring unlabelled populations. This suggested that transfer of particles either directly or indirectly through release into the medium is not occurring at a population level. This absence of apparent secondary particle uptake may be due to the presence of protein coronas on released particles, obstructing the surface silanol groups from associating with the membrane (Foldbjerg *et al*., 2013; Zhao *et al*., 2011), which could decrease subsequent binding and cell internalization. This may represent a long‐term experimental and safety benefit ensuring limiting possible leakage of the label from the target cells to unrelated cell populations *in vivo*. Particles released *in vivo* may, furthermore, be phagocytosed by macrophages, a process typically more efficient for larger particles, such as the ones used here, than for smaller ones (Burtea *et al*., 2008). This would further reduce the amount of released particles available for secondary uptake and limit the putative unspecific labelling of surrounding tissues.

The use of MRI for cell‐based therapies has a dual purpose. Not only can it precisely image the anatomical damage site and track implanted cells but it can also evaluate the extent of the repair process at the damage site (Beckmann *et al*., 2003; Henderson *et al*., 2003). It is therefore important to analyse the extent to which implanted cell populations could be detected within anatomical structures in a realistic clinical model, such as the porcine knee model presented here, which offers dimensions in line with that of human tissue. Implantation of SiMAG‐labelled cells generated significant contrast within this system and was clearly detected against anatomical structures. The visibility threshold of SiMAG‐labelled cells using a 1.5 T scanner was found to be in agreement with the threshold established *ex vivo* (10^5^ cells labelled with 5–10 μg/ml). These values are compatible with published studies, varying from single cell detection with 11.7 T scanning and μm‐sized particles (Bulte and Kraitchman, 2004; Li *et al*., 2009) to the detection of 1 × 10^6^ cells labelled with 12 μg/ml using a 3 T machine (Chen *et al*., 2012). The results presented here thus confirm that SiMAG‐based MSC labelling can meet the technical criteria outlined for use in preclinical studies (Frank *et al*., 2004).

## 5 Conclusions

Beyond imaging, magnetic particles are widely exploited in separation techniques for cell suspensions (Plouffe *et al*., 2015). *In vitro* experiments carried out in this study confirm that their use can be applied to the spatial control of cell populations. Contactless magnetic control of cell movement can further enhance patterning and seeding procedures for both 2D culture and for 3D tissue‐engineered scaffolds (Robert *et al*., 2010a; Yanai *et al*., 2012). Although *ex vivo* models have not reported consistent magnetically‐driven migration (Schafer *et al*., 2010), possibly due to variations in the particles and magnets used, such targeting approaches may open novel therapeutic applications using permanent magnet‐, electromagnet‐ or MR‐assisted cell delivery (El Haj *et al*., 2012; Riegler *et al*., 2010; Robert *et al*., 2010a; Vaněček *et al*., 2012).

Emerging MSC therapies, such as Prochymal, currently involve the use of high cell doses (in excess of 10^8^ cells) (Hare *et al*., 2009), which may in the future be reduced through improved cell delivery strategies, such as magnetically‐assisted cell targeting, to reduce the dose needed. Careful prior assessments of the particle uptake, retention profile and biological responses associated with such strategies will be critical to ensure the safe development of enhanced targeting therapies. A recent report introducing the *in vivo* labelling of stem cells prior to their harvest and allogeneic use (Khurana *et al*., 2013) underlined the requirement to ascertain the cellular innocuousness of MPs for the targeted population. The data presented in our study support the suitability of 1 μm SiMAG superparamagnetic iron oxide particles as a possible cell tracking and cell manipulation agent for stem cell‐based therapies. Their large size and coating properties, facilitating uptake, biocompatibility and visibility for MRI, make them favourable candidates for further *in vivo* preclinical research into advanced tissue engineering approaches.

## Conflict of Interest

The authors have declared no conflict of interest.

## Supporting information

Figure S1. Long‐term marker expression in MSC cultures analysed after 14 days of particle labelling
Video S1. Time‐lapse imaging of hMSC cells incubated in the presence of SiMAG MPs

Supporting info item

Supporting info item
