# Supplementary figures and images for: Autonomous magnetic labelling of functional mesenchymal stem cells for improved traceability and spatial control in cell therapy applications
Source: J Tissue Eng Regen Med. 2016 May 6;11(8):2333–48. doi: 10.1002/term.2133 (PMC5573958; doi:10.1002/term.2133)

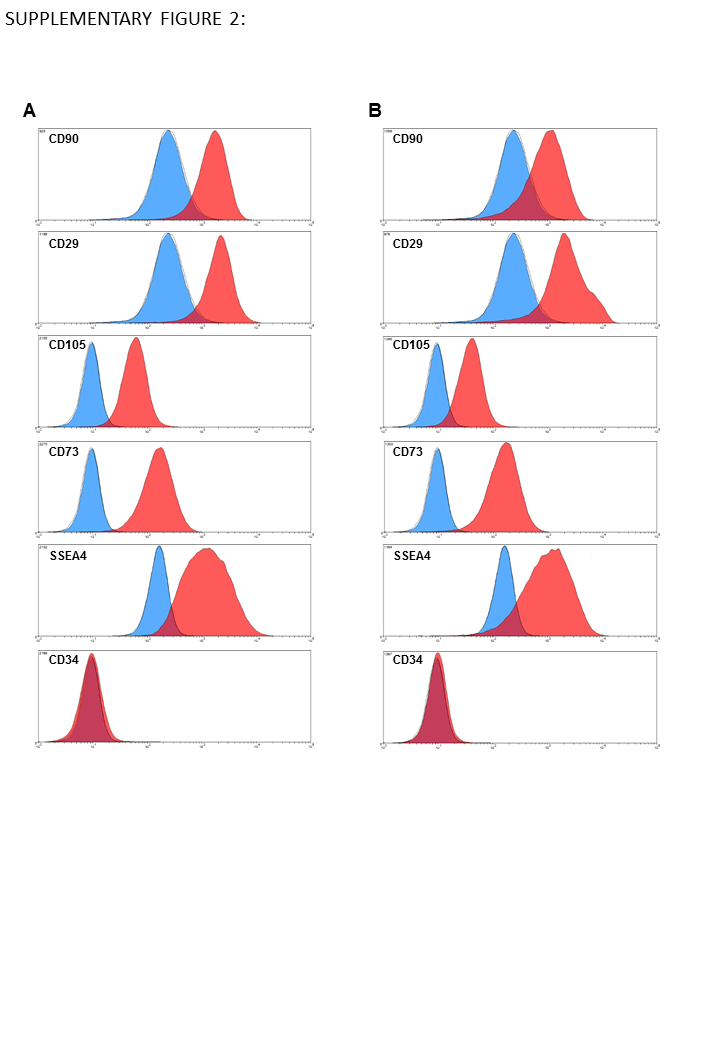

Supplement: Supplementary file 1 — Supporting info item [file TERM-11-2333-s001.tif]
